# Editorial: The impact of limited resources on antibiotic resistance in developing countries

**DOI:** 10.3389/fmicb.2026.1917235

**Published:** 2026-07-20

**Authors:** Branka Bedenić, Amir Ibrahimagić, Daniela Bandić Pavlović

**Affiliations:** 1 Clinical Department for Clinical Microbiology and Infection Prevention and Control, University of Zagreb School of Medicine, University Hospital Centre Zagreb, Zagreb, Croatia; 2 Institute for Health and Food Safety, University of Zenica, Zenica, Bosnia and Herzegovina; 3 Department for Anesthesiology and Intensive Care, University of Zagreb School of Medicine, University Hospital Centre Zagreb, Zagreb, Croatia

**Keywords:** carbapenemase, developing countries, extensively-drug resistant bacteria, low- income countries, multidrug-resistant bacteria

Antimicrobial resistance (AMR) is a top public health threat globally, claiming the lives of over 1.27 million people annually (Kapatsa et al.). It particularly affects low income countries. Africa has the world's largest mortality rate from AMR infections, which are responsible for 27 death cases per 100,000 inhabitants (Kapatsa et al.). There are estimations that it will progress to 10 million death cases annually by 2050, if no measures are undertaken (Kapatsa et al. Multidrug (MDR) and extensively-drug resistant (XDR) phenotype are frequently encountered among hospital pathogens, but lately also in the community setting. The spread of MDR bacteria is associated with additional costs and higher morbidity and mortality rates (Pendleton et al., 2013).

Developing countries in Africa and Asia and some European countries located in Southeastern Europe have very high antibiotic resistance rates, particularly high rate of ESBL and carbapenemase positivity among *Enterobacterales*. This could in part be due to lack of adequate routine microbiology diagnostic which should identify resistance traits phenotypically and using new molecular methods. Lack of staff education and resources limits the possibility for early detection which consequently enables the spread of these bad bugs. In developed countries new molecular genotyping methods such as rep-PCR, pulsed-field gel electrophoresis, multilocus-sequence typing and next generation sequencing enable rapid and accurate tracing of multidrug-resistant bacteria and prevention of large hospital outbreaks. Moreover, lapses in antibiotic prescription control, hospital hygiene and surveillance and overcrowded hospital units contribute to the spread of resistant bacteria in developing countries. The poor sanitary conditions of rural and urban families observed in certain regions in Africa and Asia may favor the transmission of bacterial infections between animals and humans, including those promoted by strains resistant to practically all available antibiotics (A WHO Practical Toolkit, 2019). CTX-M-15 which is now a dominant extended-spectrum β-lactamase (ESBL) type all over the world originated from India in the early 2000-ties. In 2008, the first case of NDM metallo-β-lactamase was reported in Sweden, but in patient previously hospitalized in India. For a long time, the majority of NDM isolates in Europe were imported from India, Pakistan, or North Africa. The data reported so far emphasize the need for One Health approach in dealing with antimicrobial resistance, in order to cope resistant bacteria.

Global inequities in the AMR burden between and within populations, are affected by the social and structural factors. Low and middle income countries experience up to 90% of total deaths from infections with resistant bacteria, high rates of infectious diseases, problems in access to healthcare and limited supply of antimicrobials (Davis et al., 2025). It is estimated that 25,000 deaths in Africa are attributable to AMR. The highest rates of AMR mortality are found in South Asia and Latin America (Davis et al., 2025). Low-income countries (LIC) are disproportionally affected due to the high burden of infectious diseases (Sullis et al., 2022). The widespread and inappropriate e use of antimicrobials in human and veterinary medicine and agriculture in LIC, coupled with inadequate waste management, generate and spread AMR (Sullis et al., 2022). Poor hygiene and lack of appropriate infection control measures are the main drivers, facilitating the spread of AMR in developing countries. COVID-19 pandemic favored the use of broad spectrum antibiotic, posing additional challenge to healthcare system (Sullis et al., 2022). The lack of public transportation in African countries also affects the access to public health institutions and antimicrobials. Women in Africa have less access to public health and antibiotics, in comparison with men, because they are usually unemployed (Davis et al., 2025).

Risk factors which facilitate the spread of MDR bacteria in LIC belong to four categories: bacterial, host-related, organizational and epidemiological (Saliba et al., 2023). In order to control the spread of MDR bacteria many countries have developed specific measures to overcome risk factors, based on surveillance cultures, the application of barriers measures, the reinforcement the hand hygiene and the implementation of contact precautions, in addition to antimicrobial stewardship program. Adopting of low-cost measures such as hand hygiene and antimicrobial stewardship was shown to reduce the risk of transmission of MDR bacteria (Saliba et al., 2023). Costly isolation protocols such as contact precautions are usually not feasible in LIC.

The specific measures that need to be undertaken to reduce the spread of MDR bacteria in low resource areas include clinician and public education, institutional treatment guidelines, surveillance cultures, antimicrobial susceptibility testing and audit feedback—mechanisms (World Health Organization, 2023). Moreover, educating and supporting healthcare workers to follow evidence-based guidelines for prescribing and administrating antibiotics is recommended by World Health Organization (WHO, 2022). Five key objectives should be fulfilled: improving awareness and understanding of AMR, strengthening knowledge via surveillance and research, reducing the incidence of infections, optimizing the use of antibiotics and ensuring sustainable investment in AMR research (WHO, 2022).

This Research Topic comprises several papers addressing different aspects of AMR in developing countries, originating from different geographic regions. The paper from Wang et al. reported the relentless spread of *mcr* genes encoding plasmid-mediated colistin resistance among extensively-drug-resistant *Escherichia coli* isolates in Chinese hospital, in addition to *bla*_NDM_ gene encoding carbapenem-resistance, *bla*_CTX − M−15_ associated with cephalosporin resistance and *aac*(3)-IVa responsible for aminoglycoside resistance. Colistin-resistance genes were carried by highly conjugative IncI2 or IncX4 plasmid types. The isolates belonged to a wide variety of sequence types: ST410, 26 ST167, ST11, ST165, and ST1266, suggesting polyclonal transmission of colistin resistance. The authors concluded that clinical invasive procedures and antibiotic exposure may have promoted the colonization and infection with resistant bacteria and that continuous monitoring of plasmid evolution is needed to overcome the spread of antimicrobial resistance.

Genomic analysis of non-typhoidal *Salmonellae* isolates from a One Health approach in Burkina Faso was described by Somda et al. One Health Approach was applied to demonstrate diffusion of *Salmonellae* in health care system, environment and agriculture. The isolates were found to possess *sul* genes for sulphonamide, *tet* for tetracycline resistance, and *cat* genes encoding chloraphenicol acetyltransferase. Interestingly, genes encoding ESBLs and carbapenemases were not found which was explained by high cost of β-lactam antibiotics which are rarely prescribed outside hospitals. Implementation of One Health Approach is necessary to improve the management and treatment of infections due to multidrug-resistant non-typhoidal *Salmonellae* and to mitigate their burden in African Sub-Saharan region.

The paper by Ammoura et al. reported high level antibiotic resistance among bacterial pathogens associated with central nervous system (CNS) infections in Nigeria. Particularly high level of AMR was reported in *S. pneumoniae* isolates with penicillin resistance reaching 100%. The authors concluded that species-level identification gaps and high AMR in CNS bacterial pathogens demand targeted microbiological-led diagnostics, including expanded cerebrospinal fluid testing and antimicrobial susceptibility guided empiric therapy in resource-limited settings.

The Turkish study (Öztürk and Kayaaslan) addressed the problem of antibiotic consumption in primary care in LIC. It was shown that it decreased in the last 10 years, particularly in pediatric population. The drop was not observed in elderly population. The high consumption of antibiotics in primary care in developing countries is the main driver for spread of AMR genes.

Analysis of the rural wastewater in India (Secker et al.) found 871 different bacteria genera, including the most important pathogens such as *Pseudomonas* spp; *Acinetobacter* spp; *Aeromonas* species, *Vibrio cholarae*, and *Bacteroides fragilis*. The resistome comprised 606 unique antimicrobial resistance genes (ARGs), with drug/biocide efflux determinants being the most prevalent, followed by macrolide and lincosamide-streptogramin B genes driven largely by 23S rRNA mutations. Carbapenemases encoding genes (*bla*_NDM_, *bla*_KPC_) and plasmid-mediated colistin resistance (*mcr*) were rarely detected. The findings of the study showed that wastewater is an important reservoir containing clinically relevant pathogens and ARG.

The study by Li et al. conducted in 22 Sub-Saharan African (SSA) countries demonstrated a decrease of the incidence of MDR and XDR tuberculosis in the majority of participating countries and the drop in the mortality rates. Progress varied substantially by region: Western SSA showed the greatest improvement, while Southern and Central SSA continued to face high burdens, with rising mortality in some areas. Strengthening diagnostics, expanding treatment access, integrating care services, and addressing key metabolic and behavioral risk factors are essential measures to accelerate tuberculosis control efforts and to reduce morbidity and mortality associated with tuberculosis.

The study carried out in Thailand (Chaudhury et al.) found high rate of ESKAPE (*Enterococcus faecium, Staphylococcus aureus, Klebsiella pneumoniae, Acinetobacter baumannii, Pseudomonas aeruginosa*, and *Enterobacter* spp.) pathogens in the untreated hospital wastewater. A high diversity of AMR was found, depending on the species. *bla*_OXA − 23_ was the most important AMR gene in *Acinetobacter baumannii*, while *bla*_NDM_ and *bla*_GES_ dominated in *Pseudomonas aeruginosa*. *bla*_KPC_ and *bla*_CTX − M−8/25_ were found in only wastewater microbiota, but absent among clinical isolates.

In conclusion, this Research Topic covers different aspects of AMR in LIC, including resistance analysis, infection control and modification of antibiotic policy in order to mitigate the burden of AMR in developing countries. Although some progress is being made in implementation of surveillance measures in developing countries, there are still problems in the implementation of concrete measures to reduce AMR rates. It is necessary to fully address the underlying structural issues that generate and promote AMR.
