# Correction: Editorial: The impact of limited resources on antibiotic resistance in developing countries

**DOI:** 10.3389/fmicb.2026.1947302

**Published:** 2026-08-10

**Authors:** Branka Bedenić, Amir Ibrahimagić, Daniela Bandić Pavlović

**Affiliations:** 1 Clinical Department for Clinical Microbiology and Infection Prevention and Control, University of Zagreb School of Medicine, University Hospital Centre Zagreb, Zagreb, Croatia; 2 Institute for Health and Food Safety, University of Zenica, Zenica, Bosnia and Herzegovina; 3 Department for Anesthesiology and Intensive Care, University of Zagreb School of Medicine, University Hospital Centre Zagreb, Zagreb, Croatia

**Keywords:** carbapenemase, developing countries, extensively-drug resistant bacteria, low- income countries, multidrug-resistant bacteria

Pendleton et al, 2013 was not cited in the article.

The citation has now been inserted in **paragraph 1, page 1** and should read:

Antimicrobial resistance (AMR) is a top public health threat globally, claiming the lives of over 1.27 million people annually (Kapatsa et al.). It particularly affects low-income countries. Africa has the world's largest mortality rate from AMR infections, which are responsible for 27 death cases per 100,000 inhabitants (Kapatsa et al.). There are estimations that it will progress to 10 million death cases annually by 2050, if no measures are undertaken (Kapatsa et al. Multidrug (MDR) and extensively-drug resistant (XDR) phenotype are frequently encountered among hospital pathogens, but lately also in the community setting. The spread of MDR bacteria is associated with additional costs and higher morbidity and mortality rates (Pendleton et al., 2013).

The reference “Pendleton, J. N., Gorman, S. P., and Gilmore, B. F. (2013). Clinical relevance of the ESKAPE pathogens. *Expert Rev. Anti Infect. Ther*. 11, 297–308. doi: 10.1586/eri.13.12” has been added to the reference list accordingly.

The original version of this article has been updated.

